# Acceleration and sprint profiles of professional male football players in relation to playing position

**DOI:** 10.1371/journal.pone.0236959

**Published:** 2020-08-06

**Authors:** José M. Oliva-Lozano, Víctor Fortes, Peter Krustrup, José M. Muyor

**Affiliations:** 1 Health Research Centre, University of Almería, Almería, Spain; 2 UD Almería, Almería, Spain; 3 Department of Sports Science and Clinical Biomechanics, Faculty of Health Sciences, University of Southern Denmark, Odense, Denmark; 4 Laboratory of Kinesiology, Biomechanics and Ergonomics (KIBIOMER Lab.), Research Central Services, University of Almería, Almería, Spain; Mary Baldwin University Murphy Deming College of Health Sciences, UNITED STATES

## Abstract

The study aims were to describe positional differences in the acceleration and sprint profiles of professional football players in match-play, and analyse start speeds required based on the intensity of accelerations and decelerations. This longitudinal study was conducted over thirteen competitive microcycles in a professional football team from LaLiga 123. Data were collected through electronic performance tracking systems. Every player was categorised based on the playing position: central defender (CD), full-back (FB), forward (FW), midfielder (MF), and wide midfielder (WMF). In respect of acceleration profile, positional differences were found for all variables (*p* < 0.05), except average magnitude of accelerations (ACC_AVG_, *p* = 0.56) and decelerations (DEC_AVG_, *p* = 0.76). The sprint profile also showed positional differences for all variables (*p* < 0.05), apart from sprint duration (*p* = 0.07). In addition, although low-intensity accelerations required significantly greater start speeds (Vo) than high-intensity accelerations in WMF (0.4 ± 0.2 km/h; *p* < 0.05) and FW (0.4 ± 0.2 km/h; *p* < 0.05), no significant differences (*p* > 0.05) were found in CD, FB, and MF. However, high-intensity decelerations were performed at significantly higher Vo than low-intensity decelerations in MF (2.65 ± 0.1 km/h; *p* < 0.05), FW (3.3 ± 0.1 km/h; *p* < 0.05), FB (3.9 ± 0.4 km/h; *p* < 0.05), WMF (4.3 ± 0.3 km/h; *p* < 0.05), and CD (4.1 ± 0.7 km/h; *p* < 0.05). Therefore, positional differences exist for most variables of the acceleration and sprint profiles. In addition, different Vo were observed between high-intensity and low-intensity accelerations as well as high-intensity and low-intensity decelerations.

## Introduction

During the past decade, there has been an increase in the literature related to athlete monitoring [1,2]. The large number of electronic performance tracking systems available on the market [2] has allowed a detailed understanding of football match demands, enabling coaches to achieve optimal training targets [1]. Football is a team sport that combines intermittent periods of high-intensity activity with longer periods of lower-intensity activity [3,4]. Professional football players cover around 10 km per match [3,5], but only 10% of the total distance is performed at high-intensity [3]. However, these high-intensity periods contribute in particular to neuromuscular fatigue, consequently increasing the risk of injury [6].

High-speed running actions, or sprints, are considered a prerequisite for successful performance in football [7,8]. In fact, sprinting skills are of prime importance in modern football [7,9]. For instance, straight sprints are the actions most frequently performed when scoring a goal [10], evading an opponent, and creating a shot on goal [11]. Thus, selection, testing, and physical conditioning of players should put emphasis on developing sprinting skills [8]. In addition, careful monitoring of these actions is necessary [9,12], taking into consideration different playing positions [13,14]. For example, a wide midfielder (WMF) may cover 294 ± 76 m of sprinting distance per match, whereas a central defender (CD) may cover 123 ± 48 m [13]. However, research on the sprint profile of professional football match-play has so far been limited [13], while different components of the sprint profile, such as sprint duration, start speed of each sprint or distance covered per sprint, have not yet been studied.

In addition, another determinant factor of football performance is the acceleration profile [7,8,13]. The acceleration profile is understood as a group of acceleration-based variables that are physically demanding [15] given the rate of change in velocity performed by the player [16]. High-intensity accelerations and decelerations have a significant impact on football players’ mechanical load [12,17] and indicators of muscle damage post-match [18]. Accelerations have a high metabolic cost [19], while decelerations increase the mechanical load [20]. Moreover, these actions are significantly associated with the neuromuscular fatigue [12] and rating of perceived exertion (RPE) [21]. For example, a previous investigation reported that the total of accelerations performed by professional football players in training sessions was significantly correlated with their session RPE [21]. Consequently, previous research suggests that understanding the acceleration profile would help to understand the potential impact that this profile might have on match performance and risk of injury [12].

Some studies have tried to individualise (based on playing position) and contextualise both acceleration [13,15,20,22] and sprint profiles [7,13,14], but most studies have analysed these profiles separately. Also, little is known, for example, about the start speed required to perform a high-intensity acceleration or deceleration [23]. The aims of this study were therefore to: 1) describe the acceleration profile of players and compare it by playing position; 2) describe the sprint profile of players and compare it by playing position; and 3) analyse the start speed (Vo) required based on the intensity of the acceleration and deceleration by playing position. Regarding the first and second aims, we hypothesised that greater positional differences may be found, particularly, between defensive and offensive positions. When it comes to the third aim, we hypothesised that high intensity accelerations and decelerations would elicit greater start speeds than low intensity accelerations or decelerations.

## Materials and methods

### Study design

The study was conducted over thirteen competitive microcycles in a professional football team from LaLiga 123. The team played one match per microcycle. The match location (home or away) alternated with each microcycle; seven matches were played away and six at home. The playing formation was 4-4-2 for all matches. Data were collected using wearable sensors (RealTrack Systems, Almería, Spain). In addition, every player was categorised based on their playing position: central defender (CD), full-back (FB), forward (FW), midfielder (MF), and wide midfielder (WMF).

### Subjects

Twenty-three professional male football players (age: 26.79 ± 3.78 years; height: 180.81 ± 6.20 cm; weight: 75.71 ± 6.88 kg; professional career: 8.40 ± 2.80 years) voluntarily took part in the study. Each player had a specific playing position: CD (n = 4), FW (n = 5), FB (n = 5), MF (n = 5) and WMF (n = 4). Given the very different nature of goalkeeping, this position was not included in the study [24]. Additionally, only players who completed the total duration of the match were analysed. Consequently, although a total of 13 matches were analyzed, not all the players from the sample participated in all the matches. The club allowed the research team to access players’ data and informed consent was provided. The study was conducted ethically according to Declaration of Helsinki and it was approved by the Bioethics Committee at the University of Almeria.

### Procedures

Data were collected using WIMU Pro 10 Hz global positioning system (GPS) devices (RealTrack Systems, Almería, Spain). This device also contains inertial sensors (four 3D accelerometers, three 3D gyroscopes, one 3D magnetometer and one barometer), which collected data at 100Hz. The validity and reliability of this device has been analysed for the collection of time-motion variables and is considered a suitable instrument for this purpose in football [25]. Regarding the validity of the device, the total bias in mean velocity measurement was between 1.18 and 1.32 km/h while the bias in distance was between 2.32 and 4.32 m [25]. In addition, good inter-unit and intra-unit reliability was reported (intraclass correlation coefficients > 0.93) [25]. The devices were calibrated according to the manufacturer’s instructions before the start of each match. All the devices were placed in the Smart Station (RealTrack Systems, Almería, Spain). First, the battery of the devices had to be fully charged. Then, a flat surface was found without any nearby magnetic devices in order to turn on the devices. After 60 seconds, the recording button was pressed. Once the calibration procedure was complete, the devices were placed in a vertical position in the back pocket of a chest vest (Rasán, Valencia, Spain).

The devices were placed in the Smart Station (RealTrack Systems, Almería, Spain) at the end of the match in order to transfer the data to the SPro software (RealTrack Systems, Almería, Spain). This software reported a database with the performance variables which were categorised into the acceleration and sprint profile as indicated in Table 1. In addition, the start speed of the action (Vo) for each high or low-intensity acceleration and deceleration was calculated in order to investigate the third aim of this study. This variable (i.e., Vo) was obtained from the “Sprint Extended” section of the “Intervals Pro” report, which was created by SPro (RealTrack Systems, Almería, Spain).

**Table 1:** Description of acceleration and sprint profile variables.

Profile	Variable	Definition
Acceleration	ACC_DIS_	Total distance covered by accelerations (m)
	DEC_DIS_	Total distance covered by decelerations (m)
	ACC_LOW_	Total number of low-intensity accelerations (below 3 m/s^2^)
	ACC_HIGH_	Total number of high-intensity accelerations (above 3 m/s^2^)
	DEC_LOW_	Total number of low-intensity decelerations (above -3 m/s^2^)
	DEC_HIGH_	Total number of high-intensity decelerations (below -3 m/s^2^)
	DIFF_ACDC_	ACC_HIGH_—DEC_HIGH_
	ACC_AVG_	Average magnitude of accelerations (m/s^2^)
	DEC_AVG_	Average magnitude of decelerations (m/s^2^)
	ACC_MAX_	Maximum magnitude of accelerations (m/s^2^)
	DEC_MAX_	Maximum magnitude of decelerations (m/s^2^)
Sprint	SPA	Total sprint actions (above 24 km/h)
	SPD	Total distance covered by sprinting (above 24 km/h)
	SPD_AVG_	Average distance covered per sprint (above 24 km/h)
	V_MAX_	Maximum speed reached in the match (km/h)
	Sprint time	Duration of sprint (s)

### Statistical analysis

First, descriptive statistics were produced for all variables of the acceleration and sprint profiles. Playing position was set as an independent variable. Then, the Shapiro-Wilk test was used to analyse the normality of the variables and Levene’s test for homogeneity. Parametric and non-parametric tests were used, since only ACC_DIS_, ACC_LOW_, DEC_LOW_, DIFF_ACDC_, V_MAX_ and sprint time were variables with normal distribution. On the one hand, when comparing the acceleration and sprint profiles relative to playing position, one-way analysis of variance (ANOVA) with Bonferroni post-hoc and Kruskal Wallis tests was used. On the other hand, when comparing the intensity of the accelerations (low-intensity accelerations, high-intensity accelerations, low-intensity decelerations, and high-intensity decelerations) based on start speed (Vo), the Mann-Whitney U test was used. The statistical power, which was calculated by G * Power software (Heinrich-Heine-Universität Düsseldorf, Düsseldorf, Germany), was greater than 0.85 in all the variables that were analyzed with the sample size of this study. The level of significance was set at *p* ≤ 0.05 and the statistical analysis was carried out using IBM SPSS Statistics version 25 (SPSS Inc., Chicago, IL, USA). Effect sizes (ES) were also calculated and categorised as trivial (0‒0.19), small (0.20‒0.49), moderate (0.50‒0.79) and large (0.80 or higher) effect [26].

## Results

Table 2 shows the descriptive statistics of the data collected for all variables of the acceleration profile and the positional differences. Significant positional differences (*p* < 0.05) were found for all variables, except DIFF_ACDC_ (*F*_4_ = 1.15; *p* = 0.33), ACC_AVG_ (*p* = 0.56) and DEC_AVG_ (*p* = 0.76). WMF was the position with the greatest ACC_DIS_ covered (436.5 ± 86.3 m; *F*_4_ = 13.63; *p* < 0.05; *ES* = 0.9–2.3) and resulted in a greater DEC_DIS_ covered compared to CD (104.4 ± 17.2 m; *p* < 0.05; *ES* = 1.7), FW (75.2 ± 17.2 m; *p* < 0.05; *ES* = 1.1), and MF (105.7 ± 16.9 m; *p* < 0.05; *ES* = 1.6). In addition, WMF performed greater ACC_HIGH_ than CD (8.5 ± 2.1; *p* < 0.05; *ES* = 1.3) and MF (7.8 ± 2.0; *p* < 0.05; *ES* = 1.3), greater DEC_HIGH_ than CD (13.6 ± 3.6; *p* < 0.05; *ES* = 1.1), and greater ACC_MAX_ (0.3 ± 0.1 m/s^2^; *p* < 0.05; *ES* = 0.6) and DEC_MAX_ (0.4 ± 0.2 m/s^2^; *p* < 0.05; *ES* = 0.5) than MF.

**Table 2:** Acceleration profile of professional football players and differences between playing positions.

	Position						
Variables	CD (M ± SD)	FB (M ± SD)	MF (M ± SD)	WMF (M ± SD)	FW (M ± SD)	p	ES
ACC_DIS_ (m)	290.8 ± 76.0^d^	351.3 ± 99.3^c^^d^	260.7 ± 64.1^b^^d^	436.5 ± 86.3^a^^b^^c^^e^	333.6 ±118.1^d^	0.01	0.17–2.32
DEC_DIS_ (m)	229.9 ± 43.0^d^	271.2 ± 66.3	228.7 ± 54.5^d^	334.4 ± 74.5^a^^c^^e^	259.2 ± 55.9^d^	0.01	0.03–1.72
ACC_LOW_ (total)	354.7 ± 54.5^c^	378.1 ± 45.8	405.9 ± 61.9^a^^d^^e^	343.8 ± 48.0^c^	351.7 ± 46.2^c^	0.01	0.06–1.11
ACC_HIGH_ (total)	26.5 ± 6.1^d^	30.4 ± 7.6	27.1 ± 5.5^d^	34.9 ± 6.7^a^^c^	29.9 ± 9.6	0.02	0.06–1.32
DEC_LOW_ (total)	355.9 ± 46.9	371.6 ± 45.9	387.4 ± 59.0^d^^e^	343.5 ± 15.8^c^	340.6 ± 40.1^c^	0.01	0.06–0.92
DEC_HIGH_ (total)	50.9 ± 8.6^d^	54.1 ± 13.3	54.8 ± 12.4	64.5 ± 15.8^a^	55.2 ± 12.1	0.02	0.04–1.07
DIFF_ACDC_ (total)	-24.4 ± 7.8	-23.7 ± 11.0	-27.7 ± 11.6	-29.5 ± 13.5	-25.3 ± 10.0	0.33	0.07–0.47
ACC_AVG_ (m/s^2^)	0.6 ± 0.1	0.6 ± 0.1	0.6 ± 0.1	0.6 ± 0.1	0.5 ± 0.1	0.56	0.03–0.22
DEC_AVG_ (m/s^2^)	-0.6 ± 0.1	-0.6 ± 0.1	-0.6 ± 0.1	-0.6 ± 0.1	-0.6 ± 0.1	0.76	0.01–0.26
ACC_MAX_ (m/s^2^)	4.5 ± 0.4	4.5 ± 0.3	4.4 ± 0.6^d^	4.70 ± 0.31^c^	4.5 ± 0.6	0.01	0.06–0.67
DEC_MAX_ (m/s^2^)	-5.7 ± 0.5^e^	-6.1 ± 0.6	-5.8 ± 0.8^d^	-6.20 ± 0.9^c^	-6.3 ± 0.9^a^	0.04	0.11–0.77

M: mean; SD: standard deviation; ES: effect size.

^a^Statistical difference to CD (p < 0.05);

^b^Statistical difference to FB (p < 0.05);

^c^Statistical difference to MF (p < 0.05);

^d^Statistical difference to WMF (p < 0.05);

^e^Statistical difference to FW (p < 0.05).

MF showed significantly greater ACC_LOW_ than WMF (62.1 ± 14.7; *F*_4_ = 5.93; *p* < 0.05; *ES* = 1.1), FW (54.1 ± 14.7; *F*_4_ = 5.93; *p* < 0.05; *ES* = 0.9), and CD (51.2 ± 14.7; *F*_4_ = 5.93; *p* < 0.05; *ES* = 0.9). MF also resulted in greater DEC_LOW_ than FW (46.8 ± 13.9; *F*_4_ = 3.96; *p* < 0.05; *ES* = 0.9) and WMF (43.9 ± 13.9; *F*_4_ = 3.96; *p* < 0.05; *ES* = 0.8). However, MF showed lower ACC_DIS_ than WMF (175.8 ± 25.5 m; *F*_4_ = 13.63; *p* < 0.05; *ES* = 2.3) and FB (90.5 ± 26.1 m; *p* < 0.05; *ES* = 1.1). In addition, FW showed higher values of DEC_MAX_ compared to CD (0.6 ± 0.2 m/s^2^; *p* < 0.05; *ES* = 0.8).

Table 3 gives the descriptive statistics for the sprint profile as well as the significant positional differences observed for all variables, apart from sprint time (*F*_4_ = 2.18; *p* = 0.07). WMF reached greater V_MAX_ compared to CD (1.4 ± 0.5 km/h; *F*_4_ = 14.47; *p* < 0.05; *ES* = 0.9), MF (3.5 ± 0.5 km/h; *F*_4_ = 14.47; *p* < 0.05; *ES* = 2.2), and FW (2.1 ± 0.5 km/h; *F*_4_ = 14.47; *p* < 0.05; *ES* = 1.2). WMF also resulted in greater SPA compared to CD (7.3 ± 1.1; *p* < 0.05; *ES* = 1.7), MF (11.3 ± 1.1; *p* < 0.05; *ES* = 2.9) and FW (7.2 ± 1.1; *p* < 0.05; *ES* = 1.8). These differences were also observed for SPD covered, which was greater in WMF than CD (189.3 ± 22.5 m; *p* < 0.05; *ES* = 2.1), MF (250.1 ± 22.1 m; *p* < 0.05; *ES* = 3.06) and FW (172.9 ± 22.5 m; *p* < 0.05; *ES* = 2.05). WMF also showed higher SPD_AVG_ than CD (4.7 ± 1.2; *p* < 0.05; *ES* = 1.46). However, MF was the position with the lowest SPA (4.6 ± 2.9; *p* < 0.05; *ES* = 1.2–2.03) and Vmax (28.5 ± 1.7 km/h; *F*_4_ = 14.47; *p* < 0.05; *ES* = 0.7–2.1).

**Table 3:** Sprint profile of professional football players and differences between playing positions.

	Position						
Variables	CD (M ± SD)	FB (M ± SD)	MF (M ± SD)	WMF (M ± SD)	FW (M ± SD)	p	ES
SPA (total)	8.6 ± 3.7^c^^d^	11.7 ± 3.9^c^	4.6 ± 2.9^a^^b^^d^^e^	15.9 ± 4.7^a^^c^^e^	8.7 ± 3.1^c^^d^	0.01	0.04–2.87
SPD (m)	148.9 ± 73.1^d^	228.3 ± 92.5^c^	88.2 ± 53.9^b^^d^^e^	338.2 ± 103.9^a^^c^^e^	165.3 ± 58.1^c^^d^	0.01	0.24–3.05
SPD_AVG_ (m)	16.9 ± 2.6^d^	19.2 ± 3.2	20.1 ± 7.7	21.6 ± 3.8^a^	19.6 ± 4.8	0.01	0.07–1.45
V_MAX_ (km/h)	30.6 ± 1.4^a^^c^^d^	30.7 ± 1.6^c^	28.5 ± 1.7^a^^b^^d^^e^	32.0 ± 1.6^a^^c^^e^	29.9 ± 2.0^c^^d^	0.01	0.07–2.16
Sprint time (s)	2.5 ± 0.3	2.8 ± 0.4	2.8 ± 1.2	3.1 ± 0.5	2.9 ± 0.7	0.07	0.03–1.45

M: mean; SD: standard deviation; ES: effect size.

^a^Statistical difference to CD (p < 0.05);

^b^Statistical difference to FB (p < 0.05);

^c^Statistical difference to MF (p < 0.05);

^d^Statistical difference to WMF (p < 0.05);

^e^Statistical difference to FW (p < 0.05).

**Fig 1:**
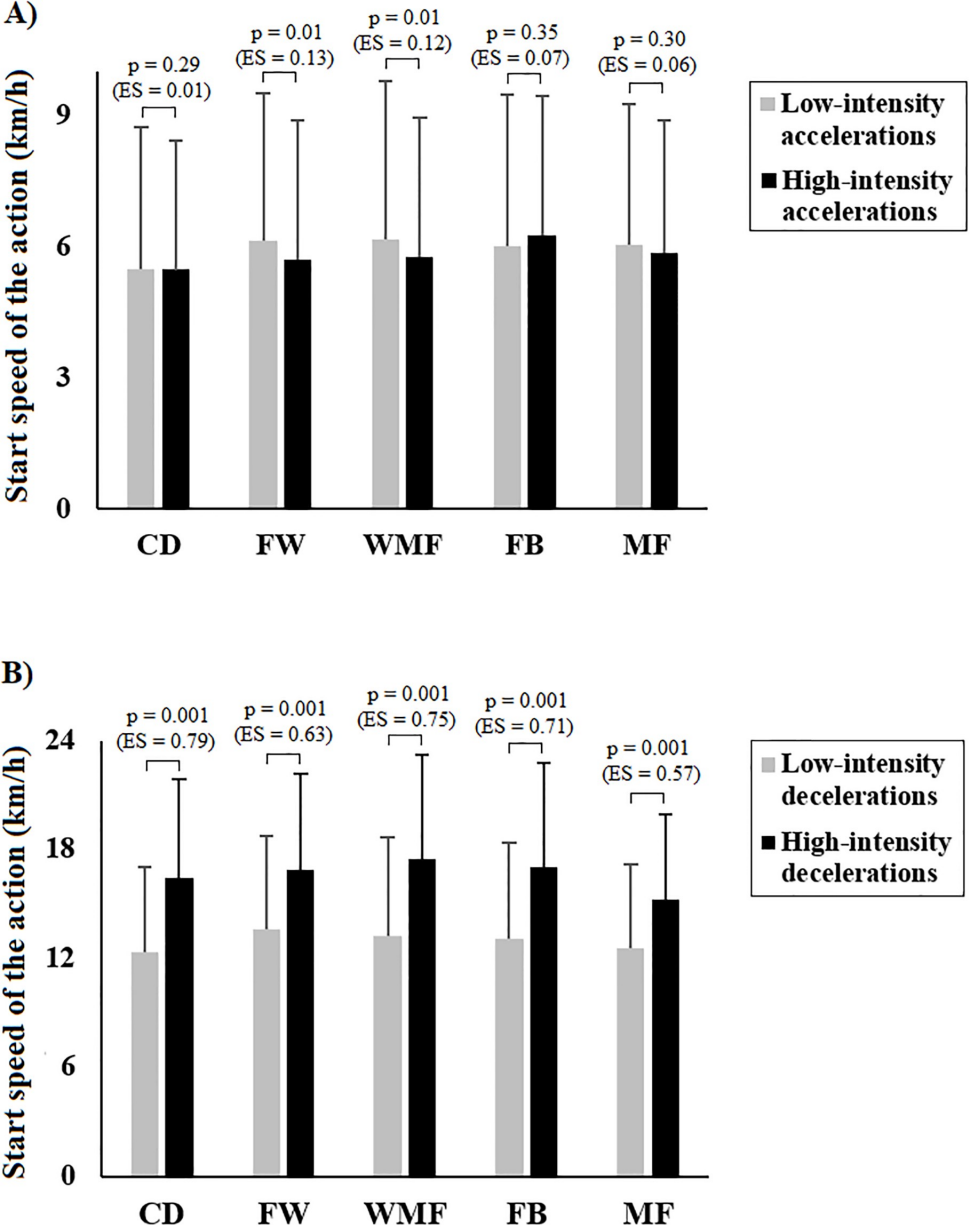
Differences on start speed of the action (Vo) based on the intensity of the accelerations/decelerations; a) Differences between low-intensity accelerations and high-intensity accelerations; b) Differences between low-intensity decelerations and high-intensity decelerations.

Fig 1 describes the Vo required to perform low-intensity accelerations, high-intensity accelerations, low-intensity decelerations, and high-intensity decelerations. On the one hand, low-intensity accelerations required significantly greater Vo than high-intensity accelerations in WMF (0.4 ± 0.2 km/h; *p* < 0.05; *ES* = 0.12) and FW (0.4 ± 0.2 km/h; *p* < 0.05; *ES* = 0.13) (Fig 1a), but no significant differences were found in CD, FB, and MF. On the other hand, high-intensity decelerations were performed at significantly higher Vo than low-intensity decelerations in MF (2.7 ± 0.1 km/h; *p* < 0.05; *ES* = 0.57), FW (3.3 ± 0.1 km/h; *p* < 0.05; *ES* = 0.64), FB (3.9 ± 0.4 km/h; *p* < 0.05; *ES* = 0.71), WMF (4.3 ± 0.3 km/h; *p* < 0.05; *ES* = 0.75), and CD (4.1 ± 0.7 km/h; *p* < 0.05; *ES* = 0.79) (Fig 1b).

## Discussion

The main purpose of this study was to describe the positional differences in the acceleration and sprint profiles of professional football players in match-play and to analyse the start speed (Vo) required based on the intensity of the acceleration and deceleration. This study showed positional differences for most variables of the acceleration and sprint profiles. Also, significant differences were observed in Vo when comparing high-intensity accelerations and high-intensity decelerations to low-intensity accelerations and low-intensity decelerations.

This study is the first to provide detailed information on the acceleration and sprint profiles of professional football players. Previous investigations [13,20,27] have described positional differences for some of the high-intensity profile variables examined in the present study but conclusions from most studies were limited because only a few variables, which are the most common in the literature, were analyzed. These studies have examined, for instance, professional Norwegian [13,20] and British [27] football teams, and found differences between playing positions for variables of the acceleration profile [13,20,27]. For example, WMF covered significantly greater ACC_DIS_ (559 ± 232 m) and DEC_DIS_ (456 ± 107 m) than MF (ACC_DIS_: 559 ± 232 m; DEC_DIS_ 360 ± 120 m) [20]. Similarly, our study showed that WMF covered significantly greater ACC_DIS_ (436.5 ± 86.3 m) and DEC_DIS_ (334.4 ± 74.5 m) than MF (ACC_DIS_: 260.7 ± 64.1 m; DEC_DIS_ 228.7 ± 54.5 m). The same study also found that FB was another position with greater ACC_DIS_ covered (714 ± 298 m) than MF (559 ± 232 m) [20]. This suggests that playing in the lateral side of the pitch in addition to the offensive and defensive roles of FB let this position cover longer ACC_DIS_ compared to central playing positions such as MF [28].

With regard to the frequency of the accelerations, another study clearly showed that the totals for ACC_HIGH_ and DEC_HIGH_ were lower than for ACC_LOW_ and DEC_LOW_ [29], which represent the nature of football as a sport that involves intermittent repeated periods of high-intensity activity [4] and thus, high neuromuscular fatigue may be developed [12]. In addition, our results support previous research reporting that WMF always performed a higher amount of ACC_HIGH_ (35 ± 5 accelerations) and DEC_HIGH_ (62 ± 9 decelerations) than CD (ACC_HIGH_: 27 ± 7 accelerations; DEC_HIGH_: 45 ± 8 decelerations) [27]. Consequently, the variable DIFF_ACDC_ also supports the same study, since the differences between playing positions were repeated once again and a higher number of DEC_HIGH_ than ACC_HIGH_ was observed in all positions [27]. However, MF performed significantly greater ACC_LOW_ and DEC_LOW_ than WMF (mean difference, ACC_LOW_: 62.1 ± 14.7 accelerations; DEC_LOW_: 43.9 ± 13.9 decelerations) and FW (mean difference, ACC_LOW_: 54.1 ± 14.7 accelerations; DEC_LOW_: 46.8 ± 13.9 decelerations) in our study, which may be explained by the fact that density increases (reduced area per player) as the ball is closer to the central zones of the pitch in match play [30]. Although WMF also had the highest DIFF_ACDC_ (-27 ± 4), this study reported that FW had the lowest DIFF_ACDC_ (-17 ± 4) [27]. Players, therefore decelerate at high-intensity more than they accelerate at high-intensity, so special focus on mechanical load indicators is recommended for strength and conditioning coaches [20]. When it comes to the magnitude of the accelerations and decelerations, the results cannot be compared to previous studies [31,32], since this is the first study to carry out this analysis in match-play. However, when analysing this variable in training contexts, differences between playing positions remained low for ACC_MAX_ (0.17 ± 0.03 m/s^2^) and DEC_MAX_ (0.26 ± 0.03 m/s^2^) [32]. Consequently, this study supports the assertion that the acceleration profile is position-dependent and that different training strategies may be adopted to improve match performance and decrease risk of injury [12].

Positional differences were found in the sprint profile, and similar conclusions were reached by previous studies that analysed some of the variables of this profile [5,15,20,33]. For example, the total of SPA performed by FW (14 ± 6 actions), FB (12 ± 5 actions) and WMF (8 ± 4 actions) was also greater than CD (5 ± 3 actions) in previous research [15]. When it comes to MF, this position showed the lowest amount of SPA not only in our study (5 ± 3 actions) but also in previous research (4 ± 4 actions) [15]. These results may be explained by the fact that this position is limited to reach high-speed actions given its tactical role (e.g., keeping ball possession, passing) and playing area [31,34,35]. Also, other studies [13,33] found that WMF covered the highest SPD (294 ± 76 m and 185 ± 23 m, respectively) whereas CD covered the lowest (123 ± 48 m and 77 ± 17 m, respectively). Thus, SPD_AVG_ was also position-dependent in this study. However, no significant differences (*p* > 0.05) were observed in a previous study in European professional players [36]. In this sense, it is interesting to note that data were collected from players who belonged to ten different teams, which might explain differences in the conclusions reached [36]. However, the same study found significant differences between playing positions when comparing V_MAX_, which is in line with our study [36]. Specifically, the V_MAX_ reached by WMF (32.0 ± 1.6 km/h) and FW (29.9 ± 2.0 km/h) was significantly higher compared to CD (30.6 ± 1.4 km/h) in our study as well as in the above-mentioned study (WMF: 32.9 ± 2.0 km/h; FW: 33.1 ± 1.9 km/h; CD: 31.7 ± 1.8 km/h) [36]. Similarly, MF showed the lowest V_MAX_ (31.0 ± 1.7 km/h) [36]. In this regard, the V_MAX_ from WMF and FW may be explained by their greater SPD_AVG_ (WMF: 21.6 ± 3.8 m; FW: 19.6 ± 4.8 m), which allow them to maximize their acceleration capacity [36]. On the contrary, MF are limited to the increase in density of players in central zones of the pitch as explained above [30]. The only variable from the sprint profile that did not show significant differences in our study was sprint time, which was in line with previous research, where the largest differences observed between positions ranged from 0.1 to 0.2 seconds [37]. Overall, these findings imply that offensive positions such as WMF and FW are subjected to the highest sprint demands, whereas CD, a defensive position characterised by short runs [13,15,33], is the least demanding position in respect of the sprint profile in professional football.

Finally, another novel finding of this study was that football players required significantly higher Vo when performing high-intensity decelerations than low-intensity decelerations across all playing positions (mean difference, CD: 4.1 ± 0.7 km/h; WMF: 4.3 ± 0.3 km/h; FB: 3.9 ± 0.4 km/h; FW: 3.3 ± 0.1 km/h; MF: 2.7 ± 0.1 km/h). However, Vo was significantly greater for low-intensity accelerations than high-intensity accelerations, and only in specific positions (WMF and FW: 0.4 ± 0.2 km/h). These results indicate that the ability to accelerate or decelerate is highly dependent on the Vo of the player, particularly, when decelerating at high-intensity (*p* < 0.01; *ES* ~ 0.69). In this regard, a recent study, which tested maximal accelerations during sprint actions at different Vo, observed a linear decrease in the maximal acceleration when Vo increased [38]. This may be explained by a typical speed-time curve from any sprint test in which the largest increase in speed is at the start of the action and then, the curve is flattened with increasing running speed [38,39]. In consequence, the acceleration capacity decreases at higher speeds [38]. Training drills could take this into consideration, since it is not only the frequency and intensity of the acceleration that is important, but also the Vo required to perform the acceleration [23]. However, it is important to understand that players occupying positions such as CD may accelerate or decelerate from lower Vo than players in other positions [23]. Since football players perform actions which start at different Vo [15], previous studies assumed that different Vo may result in different neuromuscular preload, body inclines and, therefore, different muscle group activation [38,40]. Furthermore, the players experience a massive metabolic load every time the acceleration is increased, even when speed is low [41]. Thus, when designing effective match-based drills, these profiles and positional differences may be considered for a more accurate approach to players’ performance [23]. In addition, an individualised approach to performance is needed, since these results lead to the hypothesis that, for example, a faster CD may have more ability to perform high-intensity accelerations and high-intensity decelerations than a slower player in the same position.

There are some limitations that need to be considered when interpreting the findings of this research. The data were collected using GPS technology. Only one professional football team was assessed over 13 official matches from a Spanish competition. In addition, not all the players could participate in all the matches. Another limitation of the study is that the effect of different variables such as team formation, competitive standard, or style of play on the acceleration and sprint profiles was not analysed. Future studies may consider these variables since these variables may affect match running performance [42–44]. For example, a previous study showed that teams which were characterised by possession-play covered greater distance in high-intensity running actions [44]. Also, although speed and acceleration bands/thresholds were selected according to previous studies [29,36], it is worth noting that this issue has not yet been standardised in the literature [11].

## Conclusions

The findings of this longitudinal study provide meaningful information related to the sprint and acceleration profiles of Spanish professional football match-play. This study found that positional differences exist across playing positions in both profiles, which should be considered by strength and conditioning coaches when designing effective match-based drills in training sessions. Also, special focus should be given to WMF since this position was the most demanding of the acceleration and sprint profiles. In addition, only a few studies have analysed the acceleration-velocity relationship, which is deemed important when designing training drills. Despite strength and conditioning coaches still focusing on training sprint actions starting at zero speed, most of these actions are performed at 5–6 km/h in match-play. In addition, different Vo were observed between high-intensity and low-intensity accelerations as well as high-intensity and low-intensity decelerations. This may potentially affect the neuromuscular load of the players and coaching strategies are necessary in order to maximize players’ performance. Finally, these data could also serve as a comparison source for future researchers or sports scientists and coaches from professional football teams. For example, if players decelerate at high-intensity more than they accelerate at high-intensity, coaches who choose to put more emphasis on acceleration capacity than deceleration capacity in training sessions should understand that match-play may require the opposite.

## Supporting information

S1 File (PDF)
