# Supplementary material for: Acceleration and sprint profiles of professional male football players in relation to playing position
Source: PLoS One. 2020 Aug 6;15(8):e0236959. doi: 10.1371/journal.pone.0236959 (PMC7410317; doi:10.1371/journal.pone.0236959)
Supplement: S1 File — (PDF) [file pone.0236959.s001.pdf]

SUPPORTING INFORMATION

ANOVA						
		Sum of Squares	df	Mean Square	F	Sig.
ACC_{DIS}	Between Groups	443093.356	4	110773.339	13.633	0.001
	Within Groups	934433.152	115	8125.506		
	Total	1377526.508	119			
ACC_{LOW}	Between Groups	64099.336	4	16024.834	5.933	0.001
	Within Groups	310588.589	115	2700.770		
	Total	374687.925	119			
DEC_{LOW}	Between Groups	38427.071	4	9606.768	3.959	0.005
	Within Groups	279081.729	115	2426.798		
	Total	317508.800	119			
$DIFF_{ACDC}$	Between Groups	554.765	4	138.691	1.153	0.335
	Within Groups	13831.027	115	120.270		
	Total	14385.792	119			
Sprint time	Between Groups	4.294	4	1.073	2.176	0.076
	Within Groups	56.741	115	0.493		
	Total	61.035	119			
Vmax	Between Groups	161.272	4	40.318	14.479	0.001
	Within Groups	320.232	115	2.785		
	Total	481.504	119			

DESCRIPTIVE STATISTICS AND MULTIPLE COMPARISONS

ACC_{DIS} descriptive statistics

Position	N	Mean	SD
CD	24	290.81	76.03
FW	24	333.64	118.06
WMF	24	436.52	86.32
FB	22	351.26	99.28
MF	26	260.72	64.05

ACC_{DIS} multiple comparisons

Position Groups	Mean difference	Sig.	ES
CD vs FW	-42.82	1.000	0.43
CD vs WMF	-145.70	0.001	1.79
CD vs FB	-60.44	0.250	0.69
CD vs MF	30.10	1.000	0.43
FW vs CD	42.82	1.000	0.43
FW vs WMF	-102.88	0.001	0.99
FW vs FB	-17.62	1.000	0.16
FW vs MF	72.92	0.051	0.78
WMF vs CD	145.70	0.001	1.79
WMF vs FW	102.88	0.001	0.99
WMF vs FB	85.26	0.018	0.92
WMF vs MF	175.80	0.001	2.33
FB vs CD	60.44	0.250	0.69
FB vs FW	17.62	1.000	0.16
FB vs WMF	-85.26	0.018	0.92
FB vs MF	90.54	0.007	1.10
MF vs CD	-30.10	1.000	0.43
MF vs FW	-72.92	0.051	0.78
MF vs WMF	-175.80	0.001	2.33
MF vs FB	-90.54	0.007	1.10

DEC_{DIS} descriptive statistics

Position	N	Mean	SD
CD	24	229.99	43.01
FW	24	259.22	55.96
WMF	24	334.39	74.46
FB	22	271.23	66.32
MF	26	228.66	54.54

DEC_{DIS} multiple comparisons

Position Groups	Mean difference	Sig.	ES
CD vs FW	-29.22	0.747	0.59
CD vs WMF	-104.39	0.001	1.72
CD vs FB	-41.24	0.443	0.74
CD vs MF	1.33	1.000	0.03
FW vs CD	29.22	0.747	0.59
FW vs WMF	-75.17	0.032	1.14
FW vs FB	-12.01	1.000	0.20
FW vs MF	30.56	0.693	0.55
WMF vs CD	104.39	0.001	1.72
WMF vs FW	75.17	0.032	1.14
WMF vs FB	63.16	0.088	0.89
WMF vs MF	105.73	0.001	1.63
FB vs CD	41.24	0.443	0.74
FB vs FW	12.01	1.000	0.20
FB vs WMF	-63.16	0.088	0.89
FB vs MF	42.57	0.405	0.71
MF vs CD	-1.33	1.000	0.03
MF vs FW	-30.56	0.693	0.55
MF vs WMF	-105.73	0.001	1.63
MF vs FB	-42.57	0.405	0.71

ACC_{LOW} descriptive statistics

Position	N	Mean	SD
CD	24	354.67	54.49
FW	24	351.71	46.22
WMF	24	343.79	48.03
FB	22	378.05	45.84
MF	26	405.85	61.97

ACC_{LOW} multiple comparisons

Position Groups	Mean difference	Sig.	ES
CD vs FW	2.96	1.000	0.06
CD vs WMF	10.88	1.000	0.21
CD vs FB	-23.38	1.000	0.46
CD vs MF	-51.17	0.007	0.87
FW vs CD	-2.96	1.000	0.06
FW vs WMF	7.92	1.000	0.17
FW vs FB	-26.34	0.887	0.57
FW vs MF	-54.13	0.004	0.98
WMF vs CD	-10.88	1.000	0.21
WMF vs FW	-7.92	1.000	0.17
WMF vs FB	-34.25	0.275	0.73
WMF vs MF	-62.05	0.001	1.11
FB vs CD	23.38	1.000	0.46
FB vs FW	26.34	0.887	0.57
FB vs WMF	34.25	0.275	0.73
FB vs MF	-27.80	0.674	0.50
MF vs CD	51.17	0.007	0.87
MF vs FW	54.13	0.004	0.98
MF vs WMF	62.05	0.001	1.11
MF vs FB	27.80	0.674	0.50

ACC$_{HIGH}$ descriptive statistics

Position	N	Mean	SD
CD	24	26.45	6.05
FW	24	29.91	9.56
WMF	24	34.91	6.65
FB	22	30.40	7.55
MF	26	27.07	5.46

ACC$_{HIGH}$ multiple comparisons

Position Groups	Mean difference	Sig.	ES
CD vs FW	-3.46	0.975	0.43
CD vs WMF	-8.46	0.001	1.32
CD vs FB	-3.95	0.645	0.58
CD vs MF	-0.62	1.000	0.11
FW vs CD	3.46	0.975	0.43
FW vs WMF	-5.00	0.173	0.61
FW vs FB	-0.49	1.000	0.06
FW vs MF	2.84	1.000	0.37
WMF vs CD	8.46	0.001	1.32
WMF vs FW	5.00	0.173	0.61
WMF vs FB	4.51	0.354	0.63
WMF vs MF	7.84	0.002	1.29
FB vs CD	3.95	0.645	0.57
FB vs FW	0.49	1.000	0.06
FB vs WMF	-4.51	0.354	0.63
FB vs MF	3.33	1.000	0.51
MF vs CD	0.62	1.000	0.10
MF vs FW	-2.84	1.000	0.37
MF vs WMF	-7.84	0.002	1.29
MF vs FB	-3.33	1.000	0.51

DEC_{LOW} descriptive statistics

Position	N	Mean	SD
CD	24	355.96	46.87
FW	24	340.67	40.09
WMF	24	343.50	51.22
FB	22	371.64	45.87
MF	26	387.42	59.02

DEC_{LOW} multiple comparisons

Position Groups	Mean difference	Sig.	ES
CD vs FW	15.29	1.000	0.35
CD vs WMF	12.46	1.000	0.25
CD vs FB	-15.68	1.000	0.34
CD vs MF	-31.46	0.259	0.59
FW vs CD	-15.29	1.000	0.35
FW vs WMF	-2.83	1.000	0.06
FW vs FB	-30.97	0.353	0.72
FW vs MF	-46.75	0.011	0.92
WMF vs CD	-12.46	1.000	0.25
WMF vs FW	2.83	1.000	0.06
WMF vs FB	-28.14	0.554	0.58
WMF vs MF	-43.92	0.021	0.79
FB vs CD	15.68	1.000	0.34
FB vs FW	30.97	0.353	0.72
FB vs WMF	28.14	0.554	0.58
FB vs MF	-15.79	1.000	0.30
MF vs CD	31.46	0.259	0.59
MF vs FW	46.75	0.011	0.92
MF vs WMF	43.92	0.021	0.79
MF vs FB	15.79	1.000	0.30

DEC$_{HIGH}$ descriptive statistics

Position	N	Mean	SD
CD	24	50.88	8.60
FW	24	55.25	12.08
WMF	24	64.46	15.83
FB	22	54.14	13.27
MF	26	54.77	12.39

DEC$_{HIGH}$ multiple comparisons

Position Groups	Mean difference	Sig.	ES
CD vs FW	-4.38	1.000	0.42
CD vs WMF	-13.58	0.011	1.07
CD vs FB	-3.26	1.000	0.29
CD vs MF	-3.89	1.000	0.36
FW vs CD	4.38	1.000	0.42
FW vs WMF	-9.21	0.586	0.65
FW vs FB	1.11	1.000	0.09
FW vs MF	0.48	1.000	0.04
WMF vs CD	13.58	0.011	1.07
WMF vs FW	9.21	0.586	0.65
WMF vs FB	10.32	0.247	0.70
WMF vs MF	9.69	0.291	0.68
FB vs CD	3.26	1.000	0.29
FB vs FW	-1.11	1.000	0.09
FB vs WMF	-10.32	0.247	0.70
FB vs MF	-0.63	1.000	0.05
MF vs CD	3.89	1.000	0.36
MF vs FW	-0.48	1.000	0.04
MF vs WMF	-9.69	0.291	0.68
MF vs FB	0.63	1.000	0.05

$DIFF_{ACDC}$ descriptive statistics

Position	N	Mean	SD
CD	24	-24.42	7.79
FW	24	-25.33	10.04
WMF	24	-29.54	13.51
FB	22	-23.73	11.04
MF	26	-27.69	11.59

$DIFF_{ACDC}$ multiple comparisons

Position Groups	Mean difference	Sig.	ES
CD vs FW	0.92	1.000	0.10
CD vs WMF	5.13	1.000	0.46
CD vs FB	-0.69	1.000	0.07
CD vs MF	3.28	1.000	0.33
FW vs CD	-0.92	1.000	0.10
FW vs WMF	4.21	1.000	0.35
FW vs FB	-1.61	1.000	0.15
FW vs MF	2.36	1.000	0.22
WMF vs CD	-5.13	1.000	0.46
WMF vs FW	-4.21	1.000	0.35
WMF vs FB	-5.81	0.751	0.47
WMF vs MF	-1.85	1.000	0.15
FB vs CD	0.69	1.000	0.07
FB vs FW	1.61	1.000	0.15
FB vs WMF	5.81	0.751	0.47
FB vs MF	3.97	1.000	0.35
MF vs CD	-3.28	1.000	0.33
MF vs FW	-2.36	1.000	0.22
MF vs WMF	1.85	1.000	0.15
MF vs FB	-3.97	1.000	0.35

ACC_{AVG} descriptive statistics

Position	N	Mean	SD
CD	24	0.56	0.06
FW	24	0.54	0.07
WMF	24	0.55	0.07
FB	22	0.55	0.06
MF	26	0.56	0.10

ACC_{AVG} multiple comparisons

Position Groups	Mean difference	Sig.	ES
CD vs FW	0.01	0.556	0.18
CD vs WMF	0.01	0.556	0.08
CD vs FB	0.00	0.556	0.06
CD vs MF	-0.01	0.556	0.08
FW vs CD	-0.01	0.556	0.18
FW vs WMF	-0.01	0.556	0.10
FW vs FB	-0.01	0.556	0.13
FW vs MF	-0.02	0.556	0.22
WMF vs CD	-0.01	0.556	0.08
WMF vs FW	0.01	0.556	0.10
WMF vs FB	0.00	0.556	0.03
WMF vs MF	-0.01	0.556	0.14
FB vs CD	0.00	0.556	0.06
FB vs FW	0.01	0.556	0.13
FB vs WMF	0.00	0.556	0.03
FB vs MF	-0.01	0.556	0.13
MF vs CD	0.01	0.556	0.08
MF vs FW	0.02	0.556	0.22
MF vs WMF	0.01	0.556	0.14
MF vs FB	0.01	0.556	0.13

DEC_{AVG} descriptive statistics

Position	N	Mean	SD
CD	24	-0.60	0.06
FW	24	-0.58	0.08
WMF	24	-0.60	0.08
FB	22	-0.60	0.07
MF	26	-0.60	0.10

DEC_{AVG} multiple comparisons

Position Groups	Mean difference	Sig.	ES
CD vs FW	-0.01	0.756	0.21
CD vs WMF	0.01	0.756	0.08
CD vs FB	0.00	0.756	0.06
CD vs MF	0.00	0.756	0.01
FW vs CD	0.01	0.756	0.21
FW vs WMF	0.02	0.756	0.26
FW vs FB	0.01	0.756	0.15
FW vs MF	0.02	0.756	0.17
WMF vs CD	-0.01	0.756	0.08
WMF vs FW	-0.02	0.756	0.26
WMF vs FB	-0.01	0.756	0.13
WMF vs MF	0.00	0.756	0.05
FB vs CD	0.00	0.756	0.06
FB vs FW	-0.01	0.756	0.15
FB vs WMF	0.01	0.756	0.13
FB vs MF	0.00	0.756	0.05
MF vs CD	0.00	0.756	0.01
MF vs FW	-0.02	0.756	0.17
MF vs WMF	0.00	0.756	0.05
MF vs FB	0.00	0.756	0.05

ACC_{MAX} descriptive statistics

Position	N	Mean	SD
CD	24.00	4.46	0.40
FW	24.00	4.53	0.62
WMF	24.00	4.70	0.31
FB	22.00	4.48	0.36
MF	26.00	4.39	0.63

ACC_{MAX} multiple comparisons

Position Groups	Mean difference	Sig.	ES
CD vs FW	-0.07	1.000	0.14
CD vs WMF	-0.24	0.267	0.67
CD vs FB	-0.02	1.000	0.06
CD vs MF	0.07	1.000	0.13
FW vs CD	0.07	1.000	0.14
FW vs WMF	-0.16	1.000	0.34
FW vs FB	0.05	1.000	0.10
FW vs MF	0.14	1.000	0.23
WMF vs CD	0.24	0.267	0.67
WMF vs FW	0.16	1.000	0.34
WMF vs FB	0.22	0.530	0.64
WMF vs MF	0.31	0.001	0.61
FB vs CD	0.02	1.000	0.06
FB vs FW	-0.05	1.000	0.10
FB vs WMF	-0.22	0.530	0.64
FB vs MF	0.09	0.778	0.18
MF vs CD	-0.07	1.000	0.13
MF vs FW	-0.14	1.000	0.23
MF vs WMF	-0.31	0.001	0.61
MF vs FB	-0.09	0.778	0.18

DEC_{MAX} descriptive statistics

Position	N	Mean	SD
CD	24.00	-5.73	0.52
FW	24.00	-6.33	0.98
WMF	24.00	-6.20	0.89
FB	22.00	-6.07	0.57
MF	26.00	-5.80	0.80

DEC_{MAX} multiple comparisons

Position Groups	Mean difference	Sig.	ES
CD vs FW	0.60	0.036	0.77
CD vs WMF	0.47	0.079	0.65
CD vs FB	0.34	0.093	0.62
CD vs MF	0.07	0.834	0.11
FW vs CD	-0.60	0.036	0.77
FW vs WMF	-0.13	0.729	0.14
FW vs FB	-0.27	0.706	0.33
FW vs MF	-0.53	0.119	0.60
WMF vs CD	-0.47	0.079	0.65
WMF vs FW	0.13	0.729	0.14
WMF vs FB	-0.13	0.969	0.18
WMF vs MF	-0.40	0.046	0.47
FB vs CD	-0.34	0.093	0.62
FB vs FW	0.27	0.706	0.33
FB vs WMF	0.13	0.969	0.18
FB vs MF	-0.27	0.056	0.38
MF vs CD	-0.07	0.834	0.11
MF vs FW	0.53	0.119	0.60
MF vs WMF	0.40	0.046	0.47
MF vs FB	0.27	0.056	0.38

SPA descriptive statistics

Position	N	Mean	SD
CD	24.00	8.58	3.72
FW	24.00	8.71	3.13
WMF	24.00	15.88	4.73
FB	22.00	11.68	3.97
MF	26.00	4.62	2.99

SPA multiple comparisons

Position Groups	Mean difference	Sig.	ES
CD vs FW	-0.13	1.000	0.04
CD vs WMF	-7.29	0.001	1.71
CD vs FB	-3.10	0.289	0.81
CD vs MF	3.96	0.030	1.18
FW vs CD	0.13	1.000	0.04
FW vs WMF	-7.16	0.001	1.79
FW vs FB	-2.97	0.353	0.84
FW vs MF	4.09	0.023	1.34
WMF vs CD	7.29	0.001	1.71
WMF vs FW	7.16	0.001	1.79
WMF vs FB	4.19	0.389	0.96
WMF vs MF	11.25	0.001	2.87
FB vs CD	3.10	0.289	0.81
FB vs FW	2.97	0.353	0.84
FB vs WMF	-4.19	0.389	0.96
FB vs MF	7.06	0.001	2.03
MF vs CD	-3.96	0.030	1.18
MF vs FW	-4.09	0.023	1.34
MF vs WMF	-11.25	0.001	2.87
MF vs FB	-7.06	0.001	2.03

SPD descriptive statistics

Position	N	Mean	SD
CD	24	148.99	73.08
FW	24	165.30	58.11
WMF	24	338.25	103.96
FB	22	228.27	92.52
MF	26	88.18	53.87

SPD multiple comparisons

Position Groups	Mean difference	Sig.	ES
CD vs FW	-16.31	1.000	0.24
CD vs WMF	-189.25	0.001	2.10
CD vs FB	-79.27	0.140	0.95
CD vs MF	60.82	0.209	0.95
FW vs CD	16.31	1.000	0.24
FW vs WMF	-172.95	0.001	2.05
FW vs FB	-62.97	0.763	0.82
FW vs MF	77.12	0.025	1.37
WMF vs CD	189.25	0.001	2.10
WMF vs FW	172.95	0.001	2.05
WMF vs FB	109.98	0.110	1.11
WMF vs MF	250.07	0.001	3.05
FB vs CD	79.27	0.140	0.95
FB vs FW	62.97	0.763	0.82
FB vs WMF	-109.98	0.110	1.11
FB vs MF	140.09	0.001	1.89
MF vs CD	-60.82	0.209	0.95
MF vs FW	-77.12	0.025	1.37
MF vs WMF	-250.07	0.001	3.05
MF vs FB	-140.09	0.001	1.89

SPD_{AVG} descriptive statistics

Position	N	Mean	SD
CD	24	16.91	2.58
FW	24	19.61	4.81
WMF	24	21.59	3.75
FB	22	19.15	3.18
MF	25	20.05	7.72

SPD_{AVG} multiple comparisons

Position Groups	Mean difference	Sig.	ES
CD vs FW	-2.70	0.255	0.70
CD vs WMF	-4.69	0.001	1.45
CD vs FB	-2.24	0.209	0.77
CD vs MF	-3.14	1.000	0.54
FW vs CD	2.70	0.255	0.70
FW vs WMF	-1.98	0.626	0.46
FW vs FB	0.46	1.000	0.11
FW vs MF	-0.44	1.000	0.06
WMF vs CD	4.69	0.001	1.45
WMF vs FW	1.98	0.626	0.46
WMF vs FB	2.44	0.897	0.69
WMF vs MF	1.55	0.105	0.25
FB vs CD	2.24	0.209	0.77
FB vs FW	-0.46	1.000	0.11
FB vs WMF	-2.44	0.897	0.69
FB vs MF	-0.90	1.000	0.14
MF vs CD	3.14	1.000	0.541
MF vs FW	0.44	1.000	0.06
MF vs WMF	-1.55	0.105	0.25
MF vs FB	0.90	1.000	0.14

Sprint time descriptive statistics

Position	N	Mean	SD
CD	24	2.47	0.33
FW	24	2.87	0.69
WMF	24	3.05	0.46
FB	22	2.79	0.41
MF	26	2.84	1.18

Sprint time descriptive statistics

Position Groups	Mean difference	Sig.	ES
CD vs FW	-0.41	0.165	0.75
CD vs WMF	-0.58	0.100	1.45
CD vs FB	-0.32	0.115	0.86
CD vs MF	-0.37	0.804	0.42
FW vs CD	0.41	0.165	0.75
FW vs WMF	-0.17	1.000	0.30
FW vs FB	0.09	1.000	0.15
FW vs MF	0.03	1.000	0.03
WMF vs CD	0.58	0.100	1.45
WMF vs FW	0.17	1.000	0.30
WMF vs FB	0.26	1.000	0.60
WMF vs MF	0.21	0.216	0.23
FB vs CD	0.32	0.115	0.86
FB vs FW	-0.09	1.000	0.15
FB vs WMF	-0.26	1.000	0.60
FB vs MF	-0.05	1.000	0.06
MF vs CD	0.37	0.804	0.42
MF vs FW	-0.03	1.000	0.03
MF vs WMF	-0.21	0.216	0.23
MF vs FB	0.05	1.000	0.06

V_{MAX} descriptive statistics

Position	N	Mean	SD
CD	24	30.57	1.42
FW	24	29.96	2.02
WMF	24	32.04	1.58
FB	22	30.68	1.60
MF	26	28.53	1.66

V_{MAX} multiple comparisons

Position Groups	Mean difference	Sig.	ES
CD vs FW	0.61	1.000	0.35
CD vs WMF	-1.47	0.028	0.98
CD vs FB	-0.11	1.000	0.07
CD vs MF	2.03	0.001	1.31
FW vs CD	-0.61	1.000	0.35
FW vs WMF	-2.08	0.001	1.15
FW vs FB	-0.72	1.000	0.39
FW vs MF	1.42	0.031	0.77
WMF vs CD	1.47	0.028	0.98
WMF vs FW	2.08	0.001	1.15
WMF vs FB	1.36	0.066	0.86
WMF vs MF	3.50	0.001	2.16
FB vs CD	0.11	1.000	0.07
FB vs FW	0.72	1.000	0.39
FB vs WMF	-1.36	0.066	0.86
FB vs MF	2.14	0.001	1.31
MF vs CD	-2.03	0.001	1.31
MF vs FW	-1.42	0.031	0.77
MF vs WMF	-3.50	0.001	2.16
MF vs FB	-2.14	0.001	1.31

Low-intensity accelerations and high-intensity accelerations (Vo)

Position	N	Mean Low	SD Low	Mean High	SD High	Sig.	ES
CD	24	5.49	3.23	5.46	2.96	0.29	0.01
FW	24	6.11	3.39	5.68	3.18	0.01	0.13
WMF	24	6.17	3.59	5.75	3.19	0.01	0.12
FB	22	6.01	3.45	6.24	3.20	0.35	0.07
MF	26	6.03	3.20	5.84	3.04	0.30	0.06

Low-intensity decelerations and high-intensity decelerations (Vo)

Position	N	Mean Low	SD Low	Mean High	SD High	Sig.	ES
CD	24	12.36	4.73	16.43	5.49	0.001	0.79
FW	24	13.63	5.11	16.93	5.24	0.001	0.63
WMF	24	13.24	5.47	17.49	5.75	0.001	0.76
FB	22	13.11	5.28	17.03	5.74	0.001	0.71
MF	26	12.59	4.63	15.24	4.72	0.001	0.57
